# VirBinn improves viral genome binning from metagenomic Hi-C through graph diffusion

**DOI:** 10.1093/bioinformatics/btag271

**Published:** 2026-07-07

**Authors:** Shiyuan Wang, Yuxuan Du

**Affiliations:** Department of Electrical Engineering, The University of Texas at San Antonio, One UTSA Circle, San Antonio, TX 78249, United States; Department of Electrical Engineering, The University of Texas at San Antonio, One UTSA Circle, San Antonio, TX 78249, United States

## Abstract

**Motivation:**

Metagenomic Hi-C provides *in situ* proximity signals that can improve genome binning and enable virus–host-association analysis. However, viral genome recovery remains difficult because virus–virus Hi-C contact matrices are extremely sparse. Viral genomes are small, often low-abundance, and frequently assemble into short contigs, leaving many true within-genome links unobserved and causing viral bins to fragment.

**Results:**

We present VirBinn, a graph-diffusion framework for viral binning from metagenomic Hi-C. VirBinn enhances virus–virus connectivity through two complementary mechanisms: random-walk-with-restart enhancement on the sparse virus–virus contact graph and host-guided diffusion that propagates viral seeds through the host network to infer indirect virus–virus associations. The enhanced views are integrated and clustered using Leiden community detection to produce viral metagenome-assembled genomes (vMAGs). On dataset-specific simulation benchmarks with ground truth, VirBinn consistently recovers more high-quality vMAGs than Hi-C-based and shotgun-based baselines and substantially increases the number of near-complete genomes. On four real metagenomic Hi-C datasets spanning human gut, pig gut, sheep gut (long-read assembly), and wastewater, VirBinn yields more high-completeness vMAGs under CheckV and produces bins with strong within-cluster contact support. Finally, host linkage analysis using reconstructed host MAGs reveals habitat-specific host-association patterns and plausible host taxonomic profiles.

**Availability and implementation:**

VirBinn is available at https://github.com/dyxstat/VirBinn. The scripts to reproduce the results and figures in this article are available at https://github.com/dyxstat/Reproduce_VirBinn.

## 1 Introduction

Metagenomic sequencing has transformed microbial community research by enabling direct recovery of genetic material from complex samples without cultivation (Garza and Dutilh 2015, Mallawaarachchi *et al.* 2024). However, shotgun metagenomics typically yields fragmented assemblies and, by itself, provides limited information for linking genomic fragments to their organism of origin in single-sample settings (Yu *et al.* 2018, Han *et al.* 2025). High-throughput Chromosome Conformation Capture (Hi-C) helps address this limitation by capturing *in situ* long-range DNA interactions within intact cells prior to sequencing (Beitel *et al.* 2014, Burton *et al.* 2014). In a standard Hi-C workflow, DNA is crosslinked, digested, and ligated to generate chimeric molecules that reflect 3D proximity. After alignment to metagenomic assemblies, proximity-ligation read pairs induce a contig contact matrix that quantifies physical associations among contigs (Press *et al.* 2017, Du and Sun 2022). This spatial signal complements sequence-based information and can substantially improve genome-resolved reconstruction and host–virus interaction inference in complex microbiomes.

The growing availability of metagenomic Hi-C (metaHi-C) data has motivated multiple binning approaches. Hi-C-based tools such as bin3C (DeMaere and Darling 2019), MetaTOR (Baudry *et al.* 2019), MetaCC (Du and Sun 2023), and ViralCC (Du *et al.* 2023) cluster contigs using interaction frequencies, leveraging the tendency for within-genome contacts to be denser than between-genome contacts. In parallel, shotgun-based binners, such as vRhyme (Kieft *et al.* 2022), CoCoNet (Arisdakessian *et al.* 2021), and SemiBin (Pan *et al.* 2022), use sequence composition and/or coverage coherence. Despite these advances, recovering viral genomes remains substantially more challenging than recovering bacterial genomes. Viral genomes are small, often low-abundance, and frequently assemble into short contigs, weakening both coverage-based coherence and Hi-C linkage evidence. As a result, viral contigs from the same genome are commonly split across clusters or discarded.

A central algorithmic obstacle is the sparsity of virus–virus contact matrices in metaHi-C. Because direct ligation events between fragments of a low-abundance virus are rare, the virus–virus subgraph is often poorly connected, and methods that rely primarily on direct Hi-C contact graph clustering may struggle on such sparse observations, failing to connect contigs from the same viral genome and thereby reducing recovery of high-completeness viral metagenome-assembled genomes (vMAGs) (Du *et al.* 2023). At the same time, viral contigs often retain measurable contacts to host contigs, and the host–host network typically exhibits richer structure that may provide informative paths for propagating viral signals. Our previous work, ViralCC, showed that host-associated proximity structure can provide complementary evidence for viral genome reconstruction from metagenomic Hi-C data by linking viral contigs that share associations with multiple common host contigs. However, ViralCC does not explicitly exploit connectivity among host contigs themselves. As a result, when two viral contigs are linked to different but closely connected host contigs, indirect evidence from the host graph may not be fully captured (Du *et al.* 2023). Here, we introduce VirBinn, a viral binning framework that addresses these limitations through host-guided graph diffusion. VirBinn enhances virus–virus connectivity through two complementary mechanisms: random-walk-with-restart (RWR) enhancement on the virus–virus subgraph and masked host-guided diffusion that propagates viral seeds through the host network to infer indirect virus–virus associations. The resulting enhanced graphs are integrated and clustered with Leiden community detection (Traag *et al.* 2019) to produce vMAGs. Across dataset-specific simulation benchmarks with ground truth and four real metaHi-C environments, VirBinn improves recovery of high-completeness vMAGs and yields bins with strong within-cluster contact support and biologically plausible host-association patterns.

## 2 Materials and methods

### 2.1 Overview of VirBinn

**Figure 1:**
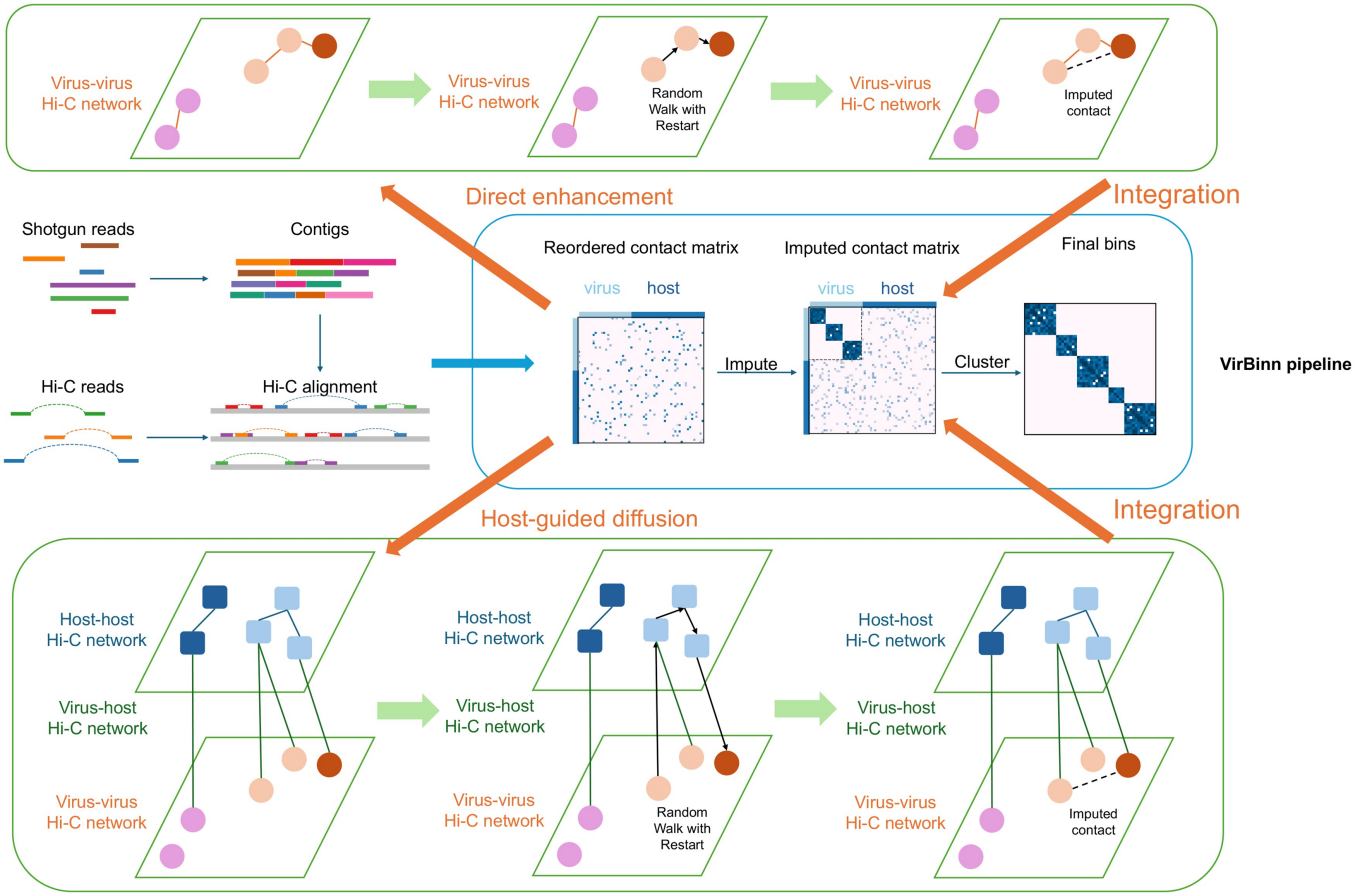
Overview of VirBinn. VirBinn normalizes the reordered Hi-C contact matrix, enhances virus–virus signals via direct random-walk-based imputation and host-guided diffusion, integrates the two enhanced graphs, and applies Leiden clustering to generate vMAGs.

VirBinn is a graph-based viral binning pipeline designed for metaHi-C datasets where virus–virus contacts are sparse and fragmented viral genomes often lack direct Hi-C links. VirBinn does not use sequence composition or abundance as explicit clustering features; instead, it operates on the Hi-C contact graph after viral contig identification. Specifically, starting from the contig contact matrix, VirBinn separates viral and non-viral contigs, normalizes contact counts using within-contig signals, and enhances virus–virus connectivity through two complementary mechanisms: (i) random-walk-based enhancement on the virus–virus subgraph to recover weak within-genome signal, and (ii) host-guided diffusion that propagates viral seeds through the host–host network to infer indirect virus–virus associations. The two enhanced views are integrated and Leiden community detection is applied to produce vMAGs (Fig. 1).

### 2.2 Real metaHi-C datasets

To benchmark VirBinn across heterogeneous microbial communities, we assembled a cross-habitat evaluation suite comprising four metaHi-C datasets spanning host-associated and environmental microbiomes: human gut (Press *et al.* 2017), pig gut (Kalmar *et al.* 2022), sheep gut (Bickhart *et al.* 2022), and wastewater (Stalder *et al.* 2019). Three datasets rely on Illumina short-read shotgun libraries paired with short-read Hi-C libraries (human gut, pig gut, and wastewater), whereas the sheep gut dataset integrates PacBio HiFi long-read shotgun sequencing with an Illumina short-read Hi-C library. Restriction enzymes used for Hi-C library construction varied by dataset: the human gut, sheep gut, and wastewater Hi-C libraries used Sau3AI and MluCI, while the pig gut library used HpyCH4IV (Table S1, available as supplementary data at *Bioinformatics* online).

The human gut dataset (Press *et al.* 2017) contains 125.4 million shotgun read pairs (37.9 Gbp) and 85.9 million Hi-C read pairs (25.9 Gbp), while the pig gut dataset (Kalmar *et al.* 2022) (HiSeq 4000) includes 86.9 million shotgun read pairs (26.1 Gbp) and 99.3 million Hi-C read pairs (29.8 Gbp). We additionally analyzed an environmental wastewater dataset (Stalder *et al.* 2019) with 269.3 million shotgun read pairs (81.3 Gbp) and 95.3 million Hi-C read pairs (28.8 Gbp). In contrast, the sheep gut metaHi-C sample (Bickhart *et al.* 2022) provides a long-read assembly setting, combining PacBio HiFi shotgun sequencing with an Illumina HiSeq 2000 Hi-C library totaling 107.7 million read pairs (32.3 Gbp). Collectively, this benchmark spans diverse hosts and environments as well as both short- and long-read assembly regimes, providing a stringent and representative testbed for viral genome binning from metaHi-C data.

### 2.3 Data preprocessing

We first standardized quality control across all metaHi-C libraries because residual adapter sequence, low-quality bases, and PCR duplicates can introduce mapping artifacts and inflate apparent contacts. All Hi-C reads were therefore preprocessed with bbduk from BBTools (v38.95) (Bushnell 2014) following a uniform cleaning protocol (Supplementary Note 1, available as supplementary data at *Bioinformatics* online). For the three short-read metaHi-C datasets, we assembled the corresponding shotgun libraries into contigs using metaSPAdes (v4.2.0) (Nurk *et al.* 2017) with default settings. For the sheep gut long-read benchmark (Bickhart *et al.* 2022), we did not reassemble the shotgun data; instead, we used the authors’ PacBio HiFi assembly generated with metaFlye (v2.9) (Kolmogorov *et al.* 2020) and downloaded it from Zenodo at https://doi.org/10.5281/zenodo.5228989 under the file “flye.v29.sheep_gut.hifi.250g.fasta.gz”. Assembly statistics for all contig sets are summarized in Table S2, available as supplementary data at *Bioinformatics* online.

Cleaned paired-end Hi-C reads were mapped to the assembled contigs with BWA-MEM (v0.7.17) (Li 2013). To accommodate proximity-ligation read structure, we disabled the default pairing behavior and retained the alignment with the lowest coordinate as the primary record using the “-5SP” option. We then filtered alignments to remove unmapped reads, secondary and supplementary alignments, and low-confidence mappings (mapping quality <30 or aligned length <30 bp). We constructed the raw contig contact map by aggregating Hi-C read pairs by their mapping destinations. Read pairs mapping to two different contigs were counted as across-contig contacts and used as the primary evidence for contig–contig proximity. Read pairs mapping to the same contig were counted as within-contig contacts and used for normalization in subsequent steps. To reduce instability driven by short contigs, we applied minimum-support filters, requiring a contig length of at least 1000 bp and at least one within-contig and one across-contig Hi-C contact by default. The resulting contact matrix has diagonal entries representing within-contig contacts and off-diagonal entries representing across-contig contacts. Unless otherwise noted, “Hi-C contacts” refer to across-contig contacts throughout this study.

### 2.4 Viral contig detection

We identified viral contigs by applying two independent callers, geNomad (v1.11.0) (Camargo *et al.* 2024) and VirSorter2 (v2.2.4) (Guo *et al.* 2021), each run with default settings. To increase specificity, we retained only contigs that were reported as viral by both tools. Contigs flagged as prophages by geNomad were subsequently removed from this viral set. All contigs not passing these criteria were designated as candidate host (non-viral) contigs.

### 2.5 The VirBinn framework

VirBinn reconstructs viral genomes from metagenomic Hi-C by combining direct signal enhancement on the sparse virus–virus contact graph and host-guided diffusion that propagates information through the typically denser host network. Starting from a raw contig contact matrix, VirBinn (i) reorders and normalizes contacts, (ii) performs random-walk-based enhancement on the virus–virus subgraph, (iii) infers indirect virus–virus associations via diffusion on a masked host graph, (iv) integrates the two enhanced views into a unified virus–virus graph, and (v) applies community detection to obtain viral bins.

#### 2.5.1 Normalization and block partitioning

Let $M\in R^{n\times n}$ be the raw Hi-C contact matrix over all contigs, where $M_{ij}$ is the number of Hi-C read pairs linking contigs *i* and *j*. Let *V* denote the set of viral contigs with |V|=k and *H* denote the remaining (non-viral) contigs with |H|=h so that n=k+h. We reorder *M* so that viral contigs appear first, yielding $M^{\text{ord}}$. To reduce biases driven by contig-specific coverage and length, we apply geometric-mean normalization using within-contig contacts:


(1) $$M_{ij}^{\prime }=\frac{M_{ij}^{\text{ord}}}{\sqrt{M_{ii}^{\text{ord}}M_{jj}^{\text{ord}}}}.$$

We then partition the normalized matrix into four blocks:


(2) $$\begin{array}{c} M^{\prime }=\left(\begin{array}{cc} M_{VV}^{\prime } & M_{VH}^{\prime }\\ M_{HV}^{\prime } & M_{HH}^{\prime } \end{array}\right), \end{array}$$

which enables separate modeling of virus–virus and virus–host/host–host interactions.

#### 2.5.2 Direct enhancement on the virus–virus graph

Because $M_{VV}^{\prime }$ is sparse, we enhance virus–virus connectivity via RWR on a row-stochastic transition matrix. We first construct a nonnegative adjacency $A_{V}$ by zeroing the diagonal of $M_{VV}^{\prime }$:


(3) $${\left(A_{V}\right)}_{ij}=\{\begin{array}{c} M_{VV,ij}^{\prime },\;i\neq j,\\ 0,\;i=j. \end{array}$$

Let $D_{V}$ be the diagonal degree matrix with ${\left(D_{V}\right)}_{ii}=\sum_{j}{\left(A_{V}\right)}_{ij}$, and define the transition matrix


(4) $$W_{V}=D_{V}^{-1}A_{V},$$

with zero-degree rows left as all zeros. Starting from $P^{\left(0\right)}=I_{k}$, we iterate


(5) $$P^{\left(t\right)}=(1-r_{1})P^{\left(t-1\right)}W_{V}+r_{1}I_{k},$$

where $r_{1}$ (default 0.5) is the restart probability. We stop when the relative change satisfies $\|P^{\left(t\right)}-P^{\left(t-1\right)}{\|}_{F}/\|P^{\left(t-1\right)}{\|}_{F}<\epsilon$ (default $\epsilon =10^{-2}$) or when a maximum iteration limit (default: 500) is reached. We then symmetrize and apply sqrtVC normalization (Rao *et al.* 2014):


(6) $$P^{\prime }=P+P^{T},\;\tilde{P}=D_{1}^{-1/2}P^{\prime }D_{1}^{-1/2},$$

where $D_{1}$ is the diagonal matrix of row sums of $P^{\prime }$.

#### 2.5.3 Host-guided diffusion for indirect virus–virus inference

To complement direct enhancement, VirBinn infers virus–virus proximity through host topology by preventing direct virus–virus transitions and allowing signal flow through virus–host and host–host edges. We define a masked adjacency


(7) $$\begin{array}{c} A_{\text{mask}}=\left(\begin{array}{cc} 0 & M_{VH}^{\prime }\\ M_{HV}^{\prime } & M_{HH}^{\prime } \end{array}\right), \end{array}$$

and convert it to a transition matrix $W_{\text{mask}}=D_{\text{mask}}^{-1}A_{\text{mask}}$ analogously to Equation (4). We seed the walk on viral nodes using


(8) $$\begin{array}{c} Q^{\left(0\right)}=\left(\begin{array}{cc} I_{k} & 0\\ 0 & 0 \end{array}\right), \end{array}$$

and iterate


(9) $$Q^{\left(t\right)}=(1-r_{2})Q^{\left(t-1\right)}W_{\text{mask}}+r_{2}Q^{\left(0\right)},$$

where $r_{2}$ (default 0.5) controls restart. To control density, we optionally sparsify after each iteration by keeping only the top τ fraction (default 20%) of nonzero entries in the *viral rows* of $Q^{\left(t\right)}$; this step is a heuristic to prevent oversmoothing and reduce computation. Convergence is assessed using the same relative Frobenius criterion as above, or the procedure is terminated upon reaching the maximum iteration limit (default: 500). In all datasets tested here, convergence was achieved well before this limit. After convergence, we extract the virus–virus block $Q_{VV}$, symmetrize, and apply sqrtVC:


(10) $$Q^{\prime }=Q_{VV}+Q_{VV}^{T},\;\tilde{Q}=D_{2}^{-1/2}Q^{\prime }D_{2}^{-1/2},$$

where $D_{2}$ is the diagonal matrix of row sums of $Q^{\prime }$.

#### 2.5.4 Graph integration and viral contig clustering

VirBinn integrates $\tilde{P}$ and $\tilde{Q}$ using an edge-budgeted sparsification rule. Let $E_{0}$ denote the set of nonzero edges in the original virus–virus contact subgraph, i.e. the off-diagonal nonzero entries of $M_{VV}^{\prime }$, and let $K=|E_{0}|$ be the corresponding edge count. We define $T_{K}(\cdot )$ for a symmetric input matrix by selecting the *K* strongest off-diagonal entries from its upper triangle, retaining the corresponding undirected edges by setting both symmetric entries to 1, and setting all remaining off-diagonal entries to 0. In this way, the number of retained inferred edges is matched to the number of edges in the original virus–virus graph. We then form


(11) $$\hat{P}=T_{K}(\tilde{P}),\;\hat{Q}=T_{K}(\tilde{Q}),\;\hat{S}=\hat{P}+\hat{Q}.$$

Thus, after sparsification, continuous edge magnitudes are no longer used directly; instead, the final integrated graph records support from the two enhancement paths. By construction, ${\hat{S}}_{ij}\in \{0,1,2\}$ indicates whether a contig pair is supported by neither path, by one of the two paths, or by both.

We apply Leiden community (Traag *et al.* 2019) detection to the weighted graph induced by $\hat{S}$ using the Reichardt–Bornholdt configuration model (Reichardt and Bornholdt 2006). The resolution parameter is optimized based on the silhouette coefficient (Rousseeuw 1987), which is a popular clustering evaluation metric without true labels by measuring the cohesion and the separation of the clusters.

### 2.6 Assessment of viral bin completeness with CheckV

We evaluated the quality of retrieved vMAGs using CheckV (v1.0.3) (Nayfach *et al.* 2021) with default settings. Because CheckV is designed to operate on a single sequence per genome, we converted each vMAG into one input sequence by concatenating its constituent contigs and using the bin ID as the FASTA header. We then ran the “end_to_end” workflow to obtain completeness estimates. CheckV determines completeness using a tiered strategy: when a vMAG matches a database reference with sufficient confidence, it estimates completeness from amino-acid-identity (AAI)-based similarity; otherwise, for more divergent or novel viruses, it falls back to an HMM-based approach that infers expected genome length from conserved viral marker genes (Nayfach *et al.* 2021).

### 2.7 Simulation-based benchmarking strategy to systematically evaluate the performance of viral contig binning

CheckV is widely used to score viral genome completeness, but it provides limited information about bin-level contamination, making it insufficient for a comprehensive assessment of vMAG quality. In addition, constructing realistic simulated benchmarks for viral metaHi-C is non-trivial because few studies have established generative models that reproduce Hi-C linkage patterns for viral contigs. To address both limitations, we adopt and extend the simulation-based benchmarking strategy introduced in ViralCC (Du *et al.* 2023), which yields (i) ground-truth labels for viral fragments and (ii) empirically consistent Hi-C contacts, without requiring *de novo* simulation of virus-specific Hi-C interactions.

#### 2.7.1 Constructing mock viral contigs with ground truth from real metaHi-C samples

Rather than starting from external reference genomes, we derive “pseudo-reference” viral genomes directly from each real dataset. Although viral assemblies are often fragmented, a subset of viral contigs can represent near-complete genomes. We therefore first ran CheckV on all detected viral contigs and selected those longer than 10 kb that CheckV labeled as “high-quality” or “complete”. These contigs were treated as putative reference viral genomes for benchmarking. We then generated mock viral fragments by splitting each putative reference genome into non-overlapping windows of length 3 kb. Terminal fragments were retained if their length exceeded 1 kb. Each resulting fragment was labeled by its source putative reference genome, providing ground-truth assignments for all mock viral contigs. To preserve the original metagenomic background, we merged these labeled mock viral contigs with the remaining non-viral contigs (treated as putative hosts) from the same sample. Finally, we aligned the original Hi-C read pairs to this mixed contig set using BWA-MEM with “-5SP” to construct a mock metaHi-C dataset whose contact patterns are inherited from the real experiment rather than synthetically generated. This procedure enables benchmarking of both Hi-C-based and shotgun-based binning tools in a controlled setting while retaining realistic experimental noise and linkage structure.

#### 2.7.2 Evaluation metrics and vMAG quality criteria

Because each mock viral contig is labeled by its source putative reference genome, we evaluated bin quality at the vMAG level using completeness and contamination defined with respect to these references. For each predicted vMAG, we summed the total contig length contributed by each reference genome and assigned the vMAG to the reference contributing the largest length, denoted L(q). Let L(r) be the length of the assigned reference genome and L(v) the total length of the vMAG. We then defined completeness as L(q)/L(r) and contamination as (L(v)−L(q))/L(v). Based on these quantities, we categorized recovered vMAGs into three tiers: near-complete (completeness ≥90%, contamination ≤10%), substantially complete (70%≤ completeness <90%, contamination ≤10%), and moderately complete (50%≤ completeness <70%, contamination ≤10%), following standard bin-quality conventions.

### 2.8 Taxonomic annotation and host linkage of vMAGs

To taxonomically classify recovered viral contigs, we applied Virgo (v1.0.0) (Riccardi *et al.* 2025). As Virgo expects per-genome (or per-contig) FASTA inputs, we split the aggregated viral contig file into individual FASTA files and ran the Virgo workflow against its reference database to obtain taxonomic assignments for each viral contig. To characterize putative hosts, we reconstructed host metagenome-assembled genomes (MAGs) from non-viral contigs using the binning and reassembly module of METAHIT (v1.0.0) (Wang *et al.* 2025) with default parameters. We then assigned taxonomy to the resulting host MAGs using GTDB-Tk (v2.4.0) (Chaumeil *et al.* 2022) by running “classify_wf” against the GTDB reference (Release 220). Finally, we linked vMAGs to host MAGs using the METAHIT MGE module with default settings, producing virus–host associations for downstream host-range analyses.

## 3 Results

### 3.1 Imputation enhancement improves viral bin recovery in simulation benchmarks

We evaluated VirBinn under controlled ground truth using dataset-specific simulation benchmarks adapted from the ViralCC evaluation strategy (Du *et al.* 2023). For each habitat, we first derived putative reference viral genomes directly from the corresponding real assembly by selecting long viral contigs (≥10 kb) that CheckV labeled as high-quality or complete (Nayfach *et al.* 2021). These contigs were then split into mock viral fragments to provide ground-truth labels, mixed back with non-viral contigs from the same sample, and paired with the original Hi-C reads remapped to this mixed contig set to preserve empirically observed linkage structure.

**Figure 2:**
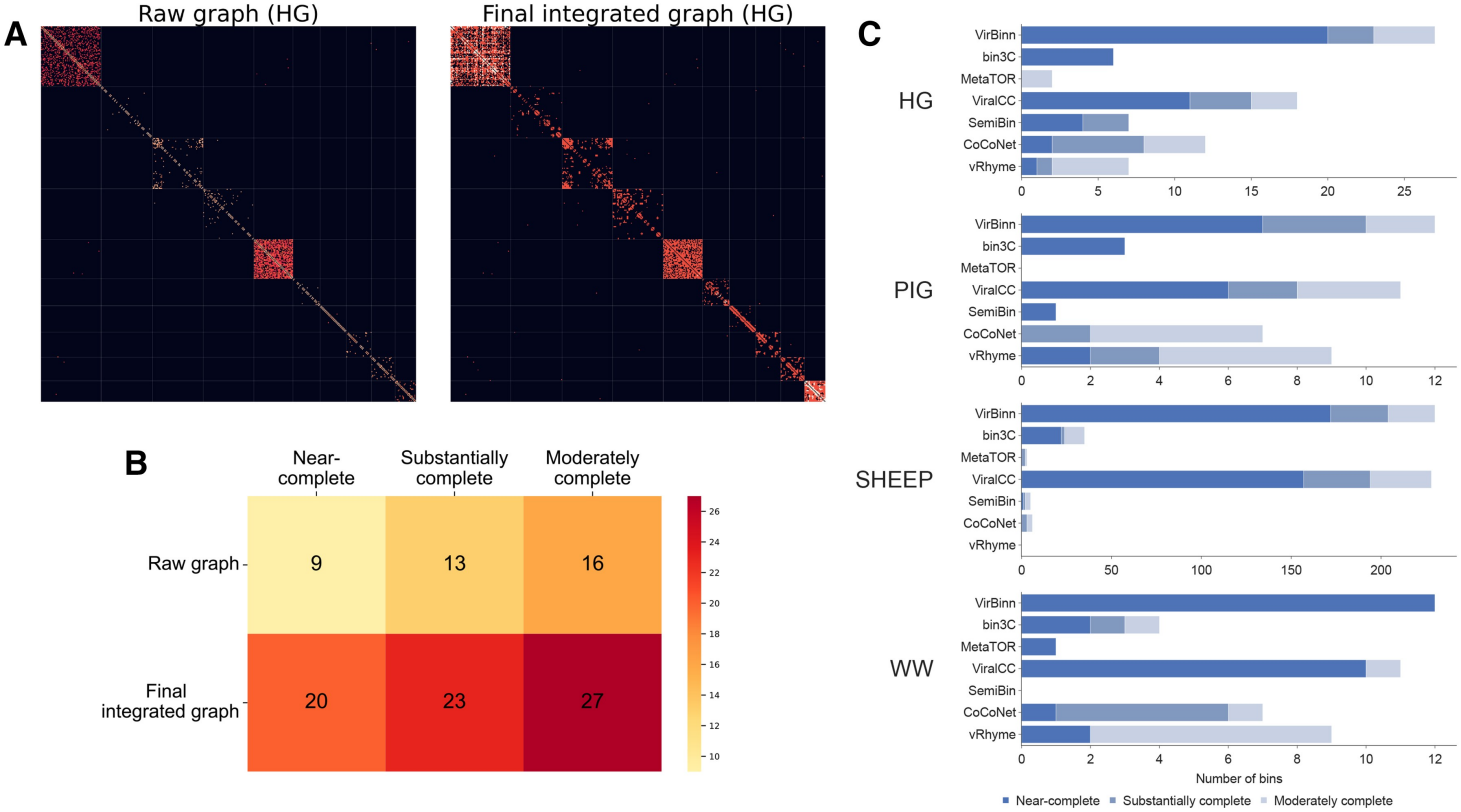
Benchmarking VirBinn performance and efficacy on simulated datasets. (A) Heatmaps of the raw and final VirBinn virus–virus graphs for the 10 most fragmented ground-truth viral genomes in the simulated human gut (HG) dataset, sorted by contig count. For visual comparability, both graphs are shown in unweighted form. The final VirBinn graph is obtained after enhancement and integration and shows more coherent within-genome block structure. (B) Numbers of recovered viral bins before and after VirBinn processing in the simulated human gut dataset. Bins are grouped as near-complete (completeness ≥90%, contamination ≤10%), substantially complete (70% ≤ completeness < 90%, contamination ≤10%), and moderately complete (50% ≤ completeness < 70%, contamination ≤10%). (C) Comparison of viral binning performance across four simulated environments (human gut, pig gut, sheep gut, and wastewater). Bar plots display the number of recovered near-complete bins, substantially complete bins, and moderately complete bins. VirBinn consistently outperforms six state-of-the-art tools (bin3C, MetaTOR, ViralCC, SemiBin, CoCoNet, and vRhyme) across all datasets and quality tiers.

We first examined how VirBinn alters the underlying virus–virus signal using the simulated human gut dataset. For the 10 ground-truth viral genomes with the greatest fragmentation, we considered the raw virus–virus Hi-C contact graph before enhancement as baseline and compared it with the VirBinn’s final integrated graph obtained after enhancement and integration (Fig. 2A). For visual comparability, both graphs are shown as unweighted adjacency matrices. In the original graph, within-genome blocks were highly sparse and often showed weak or discontinuous diagonal structure. After VirBinn processing, the corresponding blocks became more coherent and contiguous. This signal recovery translated into improved bin quality. In the human gut simulation, the number of near-complete vMAGs (completeness ≥90%, contamination ≤10%) increased from 9 to 20 after enhancement (Fig. 2B), and substantially more bins shifted into higher completeness tiers, demonstrating that mitigating contact-map sparsity is effective for reconstructing high-quality viral genomes from fragmented assemblies.

We next performed a quantitative benchmark of VirBinn on all four simulated environments (human gut, pig gut, sheep gut, and wastewater). Because ground-truth labels are available for all mock viral fragments, we evaluated each method by counting recovered high-quality vMAGs using the length-based completeness and contamination definitions in Section 2. We defined valid vMAGs as those with completeness ≥50% and contamination ≤10%, and further stratified them into near-complete (completeness ≥90%), substantially complete (70% ≤ completeness < 90%), and moderately complete (50% ≤ completeness < 70%) tiers. We compared VirBinn against three Hi-C-based binners, bin3C (v0.1.1) (DeMaere and Darling 2019), ViralCC (v1.1.0) (Du *et al.* 2023), and MetaTOR (v1.3.10; MGE module used in this study) (Bignaud *et al.* 2025), and three shotgun-based binners, vRhyme (v1.1.0) (Kieft *et al.* 2022), CoCoNet (v1.1.0) (Arisdakessian *et al.* 2021), and SemiBin (v2.2.1) (Pan *et al.* 2022). All tools were run with their default parameters. Across all simulated datasets, VirBinn consistently recovered the largest number of valid vMAGs and achieved the highest yields in each completeness tier (Fig. 2C). Specifically, in the human gut simulation, VirBinn recovered 20 near-complete vMAGs and 27 valid vMAGs overall, exceeding the next-best method by 81.8% and 50%, respectively. The same pattern was observed in the pig gut simulation. VirBinn also maintained its advantage in the long-read sheep gut and wastewater simulations, where it outperformed the best competing method, ViralCC, in both near-complete and total valid vMAG counts. Specifically, in the sheep gut simulation VirBinn assembled 172 near-complete vMAGs, surpassing ViralCC by 15 (9.6%). In the wastewater simulation, VirBinn recovered 12 valid vMAGs, all of which were near-complete, whereas ViralCC produced 10 near-complete vMAGs and one moderately complete vMAG. Collectively, these results demonstrated the effectiveness of VirBinn for reconstructing fragmented viral genomes from sparse metaHi-C signals.

### 3.2 VirBinn increases CheckV high-completeness vMAGs in real metaHi-C datasets

**Figure 3:**
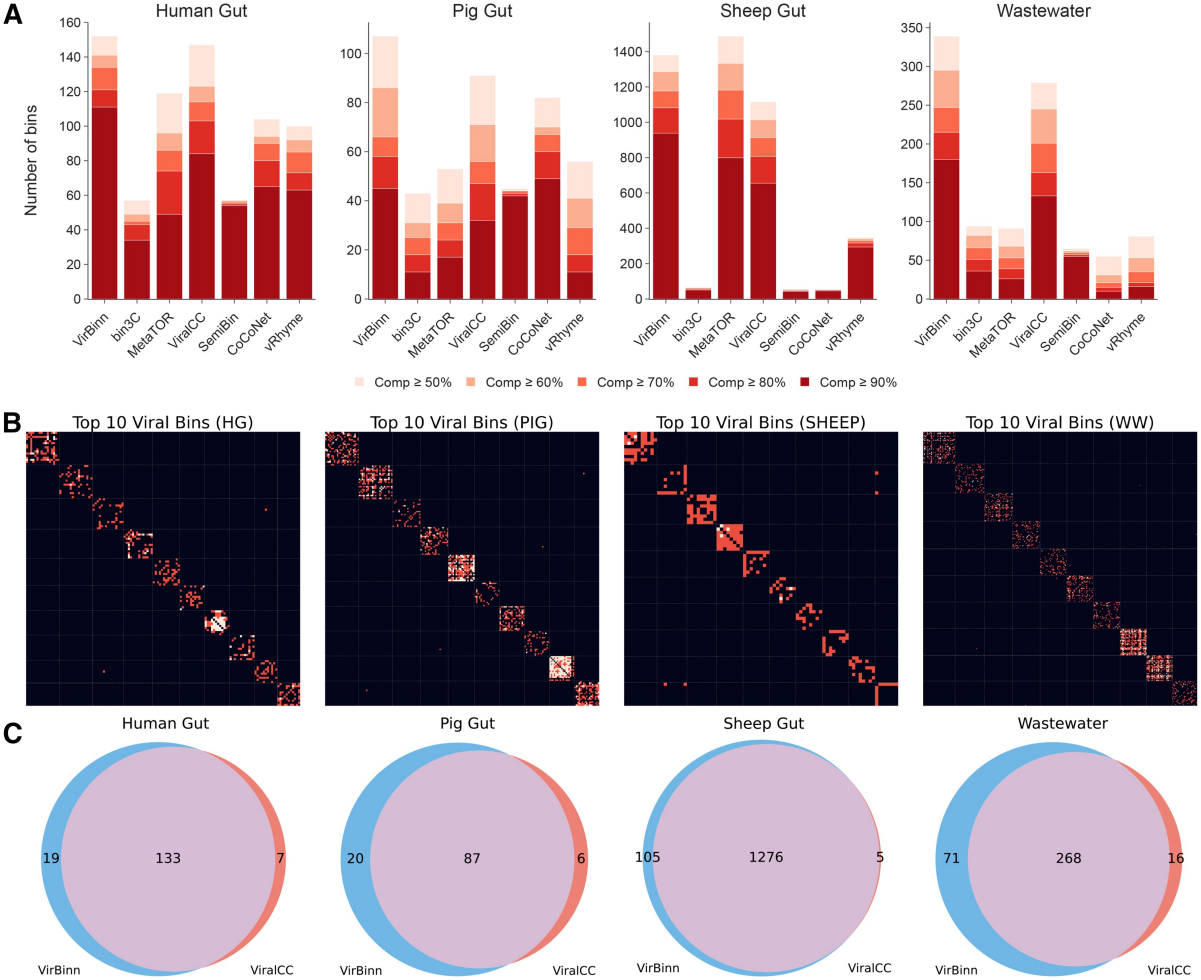
Benchmarking VirBinn performance on real metaHi-C datasets. (A) Numbers of vMAGs exceeding CheckV completeness thresholds (50%–90%) in the human gut (HG), pig gut, sheep gut, and wastewater (WW) datasets. (B) Hi-C contact heatmaps for the top 10 vMAGs, ranked by contig count, in the four real datasets. Clear diagonal patterns indicate strong within-bin contact support after VirBinn enhancement. (C) Numbers of vMAGs (CheckV completeness ≥50%) uniquely recovered by VirBinn, uniquely recovered by ViralCC, and shared by both methods across the four real datasets.

We next evaluated VirBinn on four real environments and assessed vMAG completeness using CheckV (see Section 2). Because CheckV primarily provides completeness estimates for assembled viral genomes, we compared methods by counting vMAGs exceeding stepwise completeness thresholds (50%–90%). Across all habitats, VirBinn consistently returned more high-completeness vMAGs than competing tools (Fig. 3A). Specifically, in the human gut dataset, VirBinn recovered 111 vMAGs with completeness ≥90% and 152 vMAGs with completeness ≥50%, representing improvements of 32.1% (n=27) and 3.4% (n=5) over the strongest baseline (ViralCC). In the pig gut dataset, VirBinn identified 45 vMAGs with completeness ≥90% and 107 with completeness ≥50%, exceeding ViralCC by 40.6% (n=13) and 17.6% (n=16), respectively. VirBinn also improved recovery in wastewater, yielding 180 vMAGs with completeness ≥90% and 339 vMAGs with completeness ≥50% (35.3% and 21.5% increases over ViralCC). The largest absolute gains were observed in the sheep gut long-read dataset, where VirBinn reconstructed 938 bins with completeness ≥90%, surpassing the second-best method by 139 vMAGs (17.4% increase). These results demonstrate that VirBinn improves high-completeness viral genome recovery in both host-associated and environmental settings, including long-read assemblies. Moreover, to complement the CheckV-based evaluations, we inspected the contact support underlying recovered vMAGs by plotting heatmaps of the Hi-C contact matrices for the 10 most fragmented viral bins in each dataset (Fig. 3B). These heatmaps exhibit pronounced, high-intensity diagonal blocks aligned with VirBinn clusters, indicating strong within-bin connectivity and supporting the integrity of the reconstructed viral genomes.

We further assessed overlap between VirBinn- and ViralCC-recovered vMAGs with CheckV completeness above 50% using all-versus-all Mash distances computed with Mash (Ondov *et al.* 2016) (v2.3; -s 10 000), considering two vMAGs to represent the same underlying genome when their Mash distance was below 0.01. This analysis showed that VirBinn recovered most vMAGs identified by ViralCC while also yielding many additional unique bins (Fig. 3C). To assess taxonomic breadth at the family level, we annotated these recovered vMAGs using Virgo (v1.0.0) (Riccardi *et al.* 2025) with default parameters and found that VirBinn recovered nearly all viral families identified by ViralCC while also identifying additional unique families across all four datasets (Fig. S1, available as supplementary data at *Bioinformatics* online). Together, these results demonstrate that VirBinn not only recovers most vMAGs identified by ViralCC, but also expands the taxonomic breadth of the recovered vMAG set.

Notably, VirBinn follows a virus-first workflow, in which viral contigs are first identified and subsequently clustered into vMAGs. In contrast, joint-binning strategies cluster all contigs first and separate viral bins only afterward. To assess these two workflows in practice, we compared VirBinn with joint-binning Hi-C-based baselines on the real datasets. VirBinn consistently recovered more high-completeness vMAGs than the joint-binning strategies across the four environments (Fig. S2, available as supplementary data at *Bioinformatics* online).

Finally, we compared the runtime and peak memory usage of VirBinn with those of the other binners across all four real datasets (Table S3, available as supplementary data at *Bioinformatics* online). All methods were benchmarked in the same UTSA ARC compute environment, with 80 CPU threads allocated to all tools. VirBinn completed within 45 min and used no more than 4.24 GB of memory on the human gut, pig gut, and sheep gut datasets, whereas both runtime and memory usage increased on the wastewater dataset.

### 3.3 Ablation analysis of viral RWR and host-guided diffusion

To evaluate the contributions of the two enhancement components in VirBinn, we compared the full model with two reduced configurations: viral RWR only ($\hat{P}$), derived from the random-walk-with-restart on the virus–virus subgraph, and host-guided diffusion only ($\hat{Q}$), derived from host-guided diffusion through the host network. The full model consistently outperformed both reduced configurations across the simulated datasets (Fig. S3, available as supplementary data at *Bioinformatics* online) and the real datasets (Fig. S4, available as supplementary data at *Bioinformatics* online). These results indicate that both direct viral enhancement and host-guided diffusion contribute to vMAG recovery, and that their combination yields the best overall performance. We further assessed the robustness of the integration step by replacing the default additive fusion, $\hat{S}=\hat{P}+\hat{Q}$, with a max-based alternative, $\hat{S}=\max(\hat{P},\hat{Q})$. Across the four real datasets, the two integration rules produced broadly similar results, with only small differences across datasets and completeness thresholds (Supplementary Note 2 and Fig. S5, available as supplementary data at *Bioinformatics* online).

### 3.4 Host-association patterns and host taxonomy across habitats

To place recovered vMAGs in biological context, we reconstructed host MAGs from non-viral contigs using METAHIT and linked vMAGs to candidate hosts using the METAHIT MGE module (Wang *et al.* 2025). Across all environments, a substantial fraction of vMAGs could not be assigned to any host MAG (Fig. S6A, available as supplementary data at *Bioinformatics* online), with unlinked rates ranging from 55.9% in the human gut to 87.6% in the sheep gut. This pattern is expected in metaHi-C viromics: many viruses are low-abundance, transiently associated with cells, or present as extracellular particles at the time of crosslinking, and host attribution is further limited when the true host genome is fragmented or not recovered as a high-quality MAG.

Among host-linked vMAGs, the degree of host specificity varied by habitat (Fig. S6A, available as supplementary data at *Bioinformatics* online). In the human gut, 67 vMAGs were linked to at least one host MAG, with 31 (46.3%) linked to a single host and 36 (53.7%) linked to multiple hosts. The pig gut showed a higher fraction of single-host assignments (16/27; 59.3%), whereas the sheep rumen and wastewater exhibited broader host ranges, with 71.9% (123/171) and 57.3% (51/89) of host-linked vMAGs associated with multiple hosts, respectively. Multi-host links can reflect genuine biological breadth (e.g. generalist phages or closely related host populations sharing receptors) but may also arise from ambiguity in proximity-ligation evidence when multiple related host MAGs co-occur or when linkage signal is distributed across a host community. Therefore, we interpret multi-host assignments as evidence of host-range breadth or host-context ambiguity rather than definitive proof of infection of every linked host.

Host taxonomic profiles were consistent with the expected ecology of each habitat (Fig. S6B, available as supplementary data at *Bioinformatics* online). In the gut-associated datasets, predicted hosts were dominated by *Bacillota_A* and *Bacteroidota* across both single-host and multi-host categories, matching the major bacterial lineages that structure gut ecosystems. In contrast, wastewater hosts were more taxonomically diverse and enriched for *Pseudomonadota*, reflecting the broader environmental and engineered-water microbial composition. Notably, the phylum composition differed between single-host and multi-host categories in several datasets, suggesting that host specificity is shaped not only by viral biology but also by habitat-dependent host diversity and the resolution at which host genomes can be reconstructed and distinguished.

## 4 Conclusion

In this work, we introduced VirBinn, a graph-based framework that improves viral genome reconstruction from metagenomic Hi-C by enhancing sparse virus–virus evidence before clustering. Across dataset-specific simulations with ground truth, VirBinn increased the recovery of high-quality vMAGs and shifted bins toward higher completeness tiers. On four real metaHi-C datasets spanning host-associated and environmental microbiomes, including a long-read sheep gut assembly, VirBinn consistently produced more high-completeness vMAGs under CheckV and yielded bins supported by coherent within-bin contact structure. Together, these results indicate that strengthening latent connectivity in sparse viral contact maps can materially increase the practical yield of genome-resolved viromics from metaHi-C. Notably, the method may be less reliable when viral or host assemblies are highly fragmented, host MAG recovery is poor, Hi-C sequencing depth is too shallow, or virus–host linkage patterns are ambiguous across multiple related hosts. In these settings, host-guided diffusion may propagate uncertain signals, and the resulting vMAGs should be interpreted with additional caution.

Future work will focus on extending VirBinn from genome reconstruction toward decision-ready interpretation. One direction is to develop formal confidence-aware scoring for inferred virus–virus edges and final vMAG assignments. Although the current framework distinguishes whether an inferred association is supported by direct enhancement, host-guided diffusion, or both, formal calibration of these support levels into a probabilistic confidence score remains an important direction for future work. A second direction is to incorporate complementary evidence, such as shared protein clusters, sequence composition, and sequence-based host-prediction cues, to refine vMAG annotation and better prioritize candidate virus–host relationships. Finally, adaptive graph construction strategies, including dataset-aware sparsification and automated resolution selection, may further improve robustness across sequencing depths and community complexities.

## Supplementary Material

btag271_Supplementary_Data

## Data Availability

The benchmarking datasets analyzed in this work are publicly archived in the NCBI Sequence Read Archive (SRA; http://www.ncbi.nlm.nih.gov/sra). Human gut metagenomic data are accessible under shotgun accession SRR6131123, with the corresponding Hi-C libraries available under SRR6131122 and SRR6131124. Pig gut metagenomic data are accessible under shotgun accessions ERR7197595–ERR7197599, with the corresponding Hi-C library available under ERR7197655. Sheep gut metagenomic data are accessible as a long-read assembly under the Zenodo repository https://doi.org/10.5281/zenodo.5228989 (file: flye.v29.sheep_gut.hifi.250g.fasta.gz), with the corresponding Hi-C library available under accession SRR14350344. Wastewater metagenomic data are accessible under shotgun accession SRR8239393, with the corresponding Hi-C library available under SRR8239392. All other datasets utilized by the tool are described in the article. The VirBinn software is available at https://github.com/dyxstat/VirBinn. Scripts used in this study to process the intermediate data and plot figures are available at https://github.com/dyxstat/Reproduce_VirBinn.
