# Supplementary material for: VirBinn improves viral genome binning from metagenomic Hi-C through graph diffusion
Source: Bioinformatics. 2026 Jul 7;42(Suppl 1):btag271. doi: 10.1093/bioinformatics/btag271 (PMC13340230; doi:10.1093/bioinformatics/btag271)
Supplement: btag271_Supplementary_Data [file btag271_supplementary_data.pdf]

Supplementary Information

VirBinn improves viral genome binning from metagenomic Hi-C through graph diffusion

Shiyuan Wang[1] and Yuxuan Du[1*]

[1]Department of Electrical Engineering, University of Texas at San Antonio, San Antonio, 78249, TX, USA.

*Corresponding author(s). E-mail(s): yuxuan.du@utsa.edu ;

Supplementary Notes

Supplementary Note 1: Preprocessing Parameters

Raw sequencing data was cleaned using `bbduk` (BBTools v38.95) to remove artifacts and low-quality sequences. The uniform cleaning protocol involved the following specific command-line parameters:

1. **Adapter Removal:** `ktrim=r`, `k=23`, `mink=11`, `hdist=1`
2. **Quality Trimming:** `qtrim=r`, `trimq=10`, `ftm=5`
3. **Hard Trimming:** A fixed 10 bp trim was applied to the $5'$ end of all reads.
4. **Length Filtering:** `minlen=50` (Reads shorter than 50 bp after processing were discarded).

Following these steps, FastQC (v0.11.9) was used to verify read quality.

Supplementary Note 2: Sensitivity analysis of the integration rule

To evaluate the robustness of the integration step, we compared the default additive fusion, $\hat{S} = \hat{P} + \hat{Q}$, with an alternative max-based fusion, $\hat{S} = \max(\hat{P}, \hat{Q})$, while keeping all other settings unchanged. Across the four real datasets, the two integration rules produced broadly similar results, with small differences depending on the dataset and completeness threshold (Supplementary Fig. S5). We retained the additive formulation because it preserves whether an inferred edge is supported by one or both enhancement paths, whereas the max-based rule reduces this information to a binary indicator.

Supplementary Tables

Environment	Short/Long read	Restriction enzymes	Hi-C library size
Human gut	Short	Sau3AI and MluCI	25.9 Gbp
Pig gut	Short	HpyCH4IV	29.8 Gbp
Sheep gut	Long	Sau3AI and MluCI	32.3 Gbp
Wastewater	Short	Sau3AI and MluCI	28.8 Gbp

Table S1: **Summary of the four metagenomic datasets.** The sequencing strategy is classified as either short-read or long-read metaHi-C in the 'Short/Long read' column. The specific restriction enzymes employed for Hi-C library construction are listed for each environment.

Dataset	Number of contigs	Average length (bp)	Total length (bp)
Human gut	100,214	5,058	506,843,628
Pig gut	197,665	2,979	588,749,094
Sheep gut	47,246	90,466	4,274,155,803
Wastewater	677,757	2,570	1,742,003,537

Table S2: **Contig assembly metrics by environment.** Due to the application of long-read sequencing, the sheep gut samples yielded significantly longer contigs on average.

Tool	Human Gut		Pig Gut		Sheep Gut		Wastewater	
	Time	Mem	Time	Mem	Time	Mem	Time	Mem
VirBinn	0:13:51	3.04	0:07:12	3.12	0:43:30	4.24	2:11:18	107.14
bin3C	0:01:53	0.19	0:00:44	0.19	0:01:31	0.20	0:00:34	0.19
MetaTOR	0:01:12	0.24	0:02:48	0.38	0:02:24	0.33	0:08:25	1.21
ViralCC	0:23:30	0.95	0:04:25	2.11	0:35:36	4.23	0:12:30	2.83
SemiBin	0:08:29	0.29	0:05:20	0.31	0:13:44	0.36	0:15:33	0.39
CoCoNet	0:12:48	3.87	0:17:52	3.82	0:23:29	1.66	0:18:47	1.61
vRhyme	0:06:50	0.40	0:05:10	0.41	0:06:04	0.43	0:11:20	0.41

Table S3: Runtime (h:mm:ss) and peak memory usage (GB) of VirBinn and other binning tools across the four real metaHi-C datasets.

Supplementary Figures

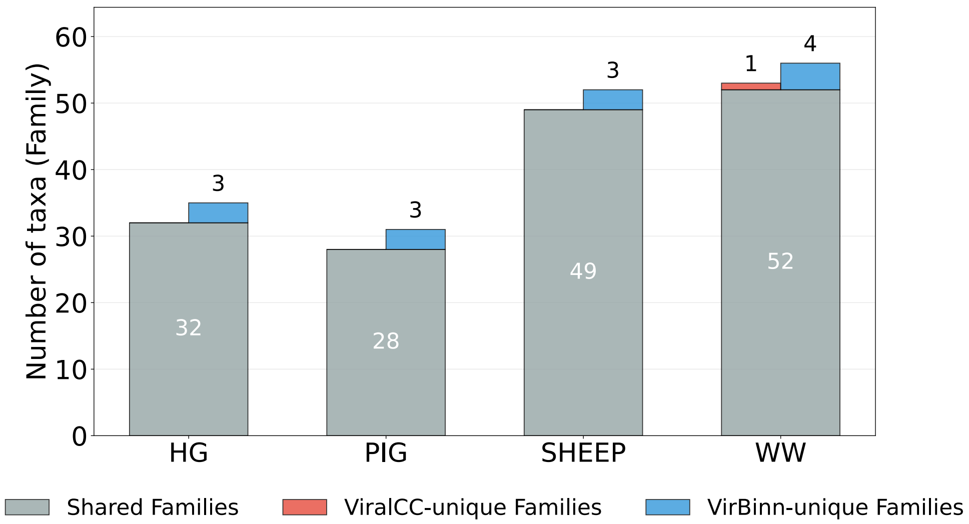

Fig. S1: **Comparison of distinct Virgo-annotated family-level taxonomic classifications derived from vMAGs with CheckV completeness above 50% recovered by VirBinn and ViralCC.** The bar plots show the shared and method-specific viral families identified by each method. Across all datasets, VirBinn recovered nearly all families identified by ViralCC while also identifying additional unique families.

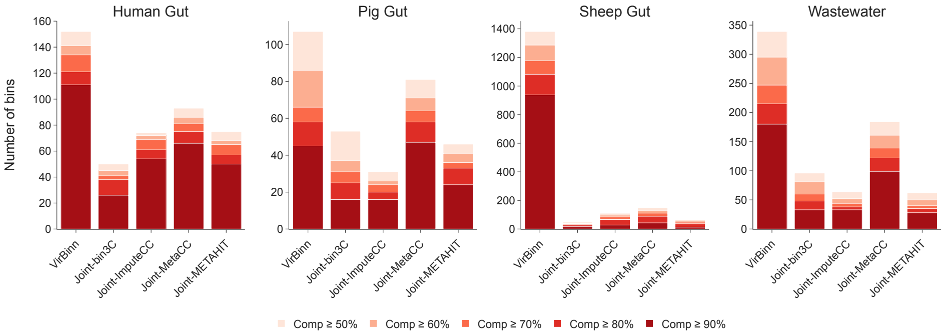

Fig. S2: **Comparison of VirBinn with joint-binning Hi-C-based baselines across four real metaHi-C datasets.** VirBinn follows a virus-first workflow, in which viral contigs are first identified and then clustered into vMAGs. Joint-bin3C, Joint-ImputeCC, Joint-MetaCC, and Joint-MetaHIT denote joint-binning baselines constructed by applying bin3C, ImputeCC, MetaCC, and MetaHIT, respectively, to all contigs first and then extracting viral bins from the resulting clusters. Bars show the numbers of vMAGs exceeding CheckV completeness thresholds in the human gut, pig gut, sheep gut, and wastewater datasets. VirBinn consistently recovered more high-completeness vMAGs than the joint-binning strategies across the four environments.

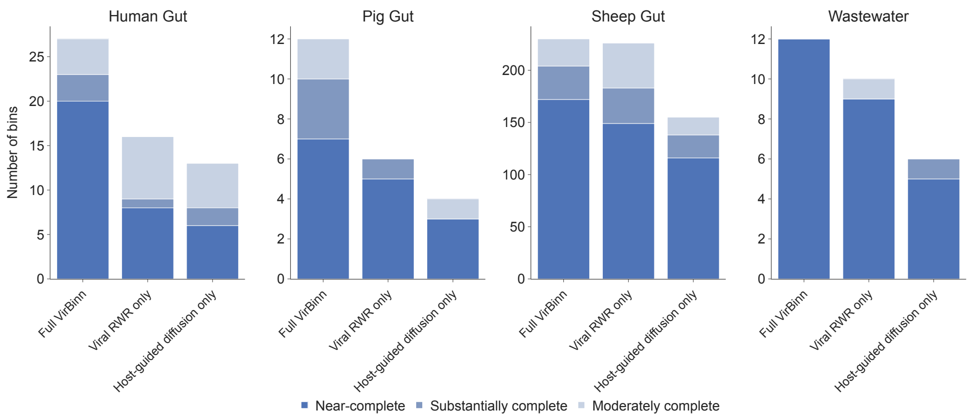

Fig. S3: **Ablation analysis of VirBinn using viral RWR-only and host-guided-diffusion-only configurations across the simulated datasets.** Bar plots indicate the total number of reconstructed vMAGs across the four simulated datasets. Bins are grouped by quality: near-complete (completeness $\geq$ 90%, contamination $\leq$ 10%), substantially complete (70% $\leq$ completeness $<$ 90%, contamination $\leq$ 10%), and moderately complete (50% $\leq$ completeness $<$ 70%, contamination $\leq$ 10%).

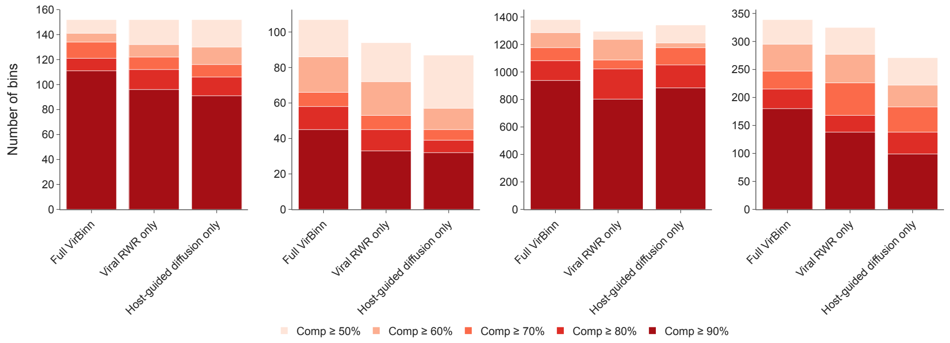

Fig. S4: **Ablation analysis of VirBinn using viral RWR-only and host-guided-diffusion-only configurations across the real datasets.** Bar plots indicate the total numbers of vMAGs exceeding CheckV completeness thresholds in real datasets. Across the human gut, pig gut, sheep gut, and wastewater datasets, the full VirBinn model consistently recovered more high-completeness vMAGs than either reduced configuration.

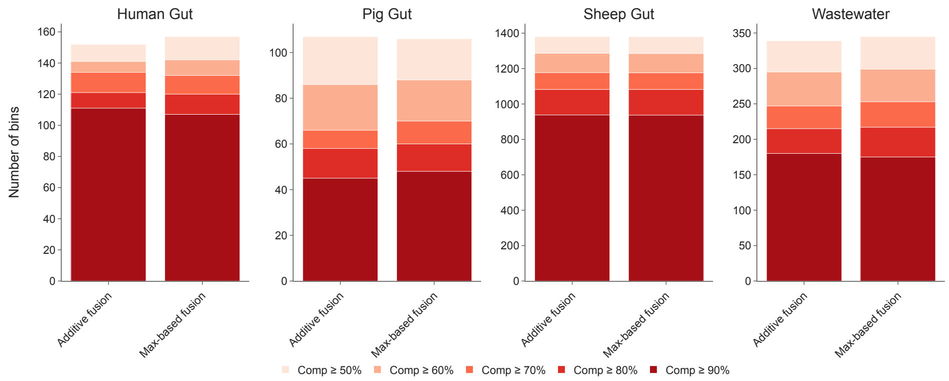

Fig. S5: **Comparison of additive and max-based graph integration in VirBinn across the four real datasets.** Bars show the numbers of vMAGs exceeding CheckV completeness thresholds under the two integration rules.

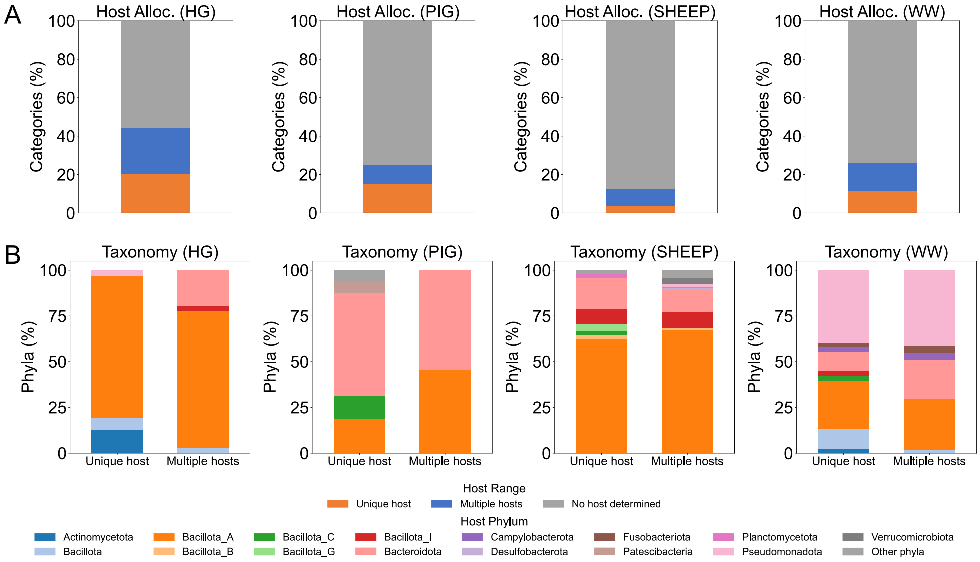

Fig. S6: **Host linkage and host taxonomy of vMAGs. A** Fractions of vMAGs that are unlinked, linked to a single host, or linked to multiple hosts based on METAHIT host MAG reconstruction and MGE linkage. **B** Phylum-level taxonomic composition of predicted hosts for vMAGs linked to a single host (left) or multiple hosts (right).
